# Physician-staffed prehospital units: a retrospective follow-up from an urban area in Scandinavia

**DOI:** 10.1186/s12245-023-00519-8

**Published:** 2023-07-14

**Authors:** Erik Strandqvist, Staffan Olheden, Anders Bäckman, Henrik Jörnvall, Denise Bäckström

**Affiliations:** 1 Capio Akutläkarbilar, Stockholm, Sweden; 2 Function Perioperative Medicine and Intensive Care, Department of Perioperative Care, Solna Karolinska University Hospital, Stockholm, Sweden; 3 Center for Resuscitation Science, Karolinska Institutet, Södersjukhuset, Stockholm, Sweden; 4 Department of Physiology and Pharmacology, Section for Anesthesia and Intensive Care Medicine, Karolinska Institutet, Stockholm, Sweden; 5 Department of Biomedical and Clinical Sciences, Linköping University, 581 83 Linköping, Sweden

**Keywords:** Prehospital, Rapid response vehicle, Scandinavia, Sweden, Trauma, Cardiac arrest

## Abstract

**Background:**

The aim of this study was to determine when and how rapid response vehicles (RRVs) make a difference in prehospital care by investigating the number and kinds of RRV assignment dispatches and the prehospital characteristics and interventions involved.

**Methods:**

This retrospective cohort study was based on data from a quality assurance system where all assignments are registered. RRV staff register every assignment directly at the site, using a smartphone, tablet, or computer. There is no mandatory information requirement or time limit for registration. The study includes data for all RRVs operating in Region Stockholm, three during daytime hours and one at night – from January 1, 2021 to December 31, 2021.

**Results:**

In 2021, RRVs in Stockholm were dispatched on 11,283 occasions, of which 3,571 (31.6%) resulted in stand-downs. In general, stand-downs were less common for older patients. The most common dispatch category was blunt trauma (1,584 or 14.0%), which accounted for the highest frequency of stand-downs (676 or 6.0%). The second most common category was cardiac arrest (1,086 or 9.6%), followed by shortness of breath (691 or 6.1%), medical not specified (N/S) (596 or 5.3%), and seizures (572 or 5.1%).

**Conclusion:**

The study findings confirm that RRVs provide valuable assistance to the ambulance service in Stockholm, especially for cardiac arrest and trauma patients. In particular, RRV personnel have more advanced medical knowledge and can administer medications and perform interventions that the regular ambulance service cannot provide.

**Supplementary Information:**

The online version contains supplementary material available at 10.1186/s12245-023-00519-8.

## Background

International and national comparative studies of prehospital care present a number of challenges, including diversity of practices, differing quality indicators, and variations in available resources. For example, available resources dictate all aspects of care, from vehicles and equipment to staffing and training [1]. While prehospital care in the Nordic countries is similar in many respects, there are also significant differences – for example, in staff training and education [2, 3].

Across the Scandinavian countries, physicians (usually anaesthesiologists) are present in prehospital settings, although the numbers vary across countries. Complementing other prehospital units, these physicians typically use cars or helicopters [3, 4]. Anaesthesiologists also work in prehospital units in other European countries and Australia [5, 6]. These units provide higher levels of medical expertise that facilitate more advanced diagnosis, intervention, and treatment [7–9]. Even if a physician does not initiate treatment on arrival, research suggests that ambulance crews value their presence, diagnostic ability, and clinical judgment [10, 11]. For these units, the most common assignment is medical etiology, followed by trauma [4].

The annual incidence of critically ill or injured patients is approximately 25–30 persons per 10,000 [4]. There is also evidence that units staffed by anaesthesiologists have significantly higher success rates for prehospital intubation [8]. One Norwegian study also reported that triage is more accurate when performed by prehospital anaesthesiologists as compared to regular ambulance crew, with reduced levels of both overtriage and undertriage [12]. Among other advantages, the presence of prehospital physicians helps to prevent unnecessary ambulance transport to hospital [12].

In Stockholm, rapid response vehicles (RRVs) have been in service since 2008. Operated by a private company (currently Capio Läkarbilar AB) [13], they are stationed at three different locations across the city. In the northern area, Nordbilen operates seven days a week from 07:00 to 21:00; in the central area, Mittbilen operates 24/7; and in the southern area, Sydbilen operates seven days a week from 07:00 to 21:00. All RRVs are dispatched by the emergency call centre (SOS Alarm AB).

Nordbilen and Sydbilen are staffed by emergency medicine physicians and an anaesthesia nurse. Mittbilen is staffed by an anaesthesiologist and a nurse specializing in prehospital care [13].

The aim of the present study was to determine when and how RRVs make a difference in prehospital care by investigating the number and kinds of RRV assignment dispatches and the prehospital characteristics and interventions involved.

## Method

### Study design and participants

This retrospective cohort study draws on data from LogEze (Fitymi AB, Bråvallagatan 6, 113 36 Stockholm, Sweden) a quality assurance system where all assignments are registered. RRV staff register every assignment directly at the site, using a smartphone, tablet, or computer. There is no mandatory information requirement or time limit for registration. The study includes data for all RRVs operating in the Stockholm Region – three during daytime hours and one at night – from January 1, 2021 to December 31, 2021. The total number of assignments was 11,455, of which 172 were excluded as multiple registrations of the same case number. As of today, the regular ambulances don’t register or report their assignments in any database and did not have access to LogEze during this study period. The study was approved by the Swedish Ethical Review Authority (number 2021–05498-01).

### Setting

Stockholm’s RRVs serve a population of approximately 2.4 million inhabitants across an area of 6,519 km^2^ [14]. The region has six main hospitals; major trauma patients are referred to the regional trauma centre. RRVs are dispatched from the dispatch centre based on a found or probable illness. Ambulance crews can also ask specifically for RRV medical assistance while caring for a patient. Prehospital Trauma Life Support (PHTLS) is used nationwide. The three main categories of healthcare workers in the prehospital ambulance service are (1) prehospital emergency nurses, who have a degree in nursing and a graduate degree in prehospital emergency care; (2) ambulance nurses, who have a degree in nursing; and (3) emergency medical technicians, whose medical training ranges from six months to two years.

### Characteristics

A detailed description can be found in Appendix 1.

### Priority

Priority 1 means *very urgent assignment, life-threatening condition*. Priority 2 means *urgent assignment, acute condition, non-life-threatening*. Priority 3 means *non-urgent assignment, non-acute condition, no impact on patient for waiting*. Priorities 4–9 indicate that the patient needs assessment for a non-urgent or non-life-threatening condition. Priorities 5 and 9 typically include a visit from a nurse or family doctor [15].

### Stand-downs

If ambulance personnel decide they do not need assistance from an RRV, they make radio contact to report the patient’s SBAR status (Situation, Background, Assessment, Recommendation). Following an ambulance report that further assistance is not needed, the RRV physician makes a decision to abort the assignment or proceed [16].

### Significant impact and deviation from guidelines

The RRV team may also report that they had a significant impact on the healthcare provided, or that their actions deviated from current guidelines or standard operating procedures for the patient in question. However, no details are provided regarding the nature of the impact or whether it affected the patient's outcome. There was only one option to choose from: whether the RRV team had a significant impact or not. Upon arriving at the scene, the physician and nurse in the RRV typically ask the ambulance crew how they can assist, and in some situations, the RRV team takes over management of the situation, while in other cases, they may only provide minor assistance.

### Statistics

The data were analysed using Microsoft Excel 2019 (Microsoft Corp, Redmond, WA, USA) and the Statistical Package for Social Sciences (SPSS) version 27 (SPSS, Chicago, III, USA). Values are presented as number and percentage of cases. Continuous data are presented as mean and standard deviation (SD). For between-group comparison, Student’s T-test was used for continuous data, and Pearson’s chi-squared test was used for categorical data frequencies. Data imputation was not used to correct for missing values. Probabilities of less than 0.05 were accepted as significant.

## Results

**Fig. 1:**
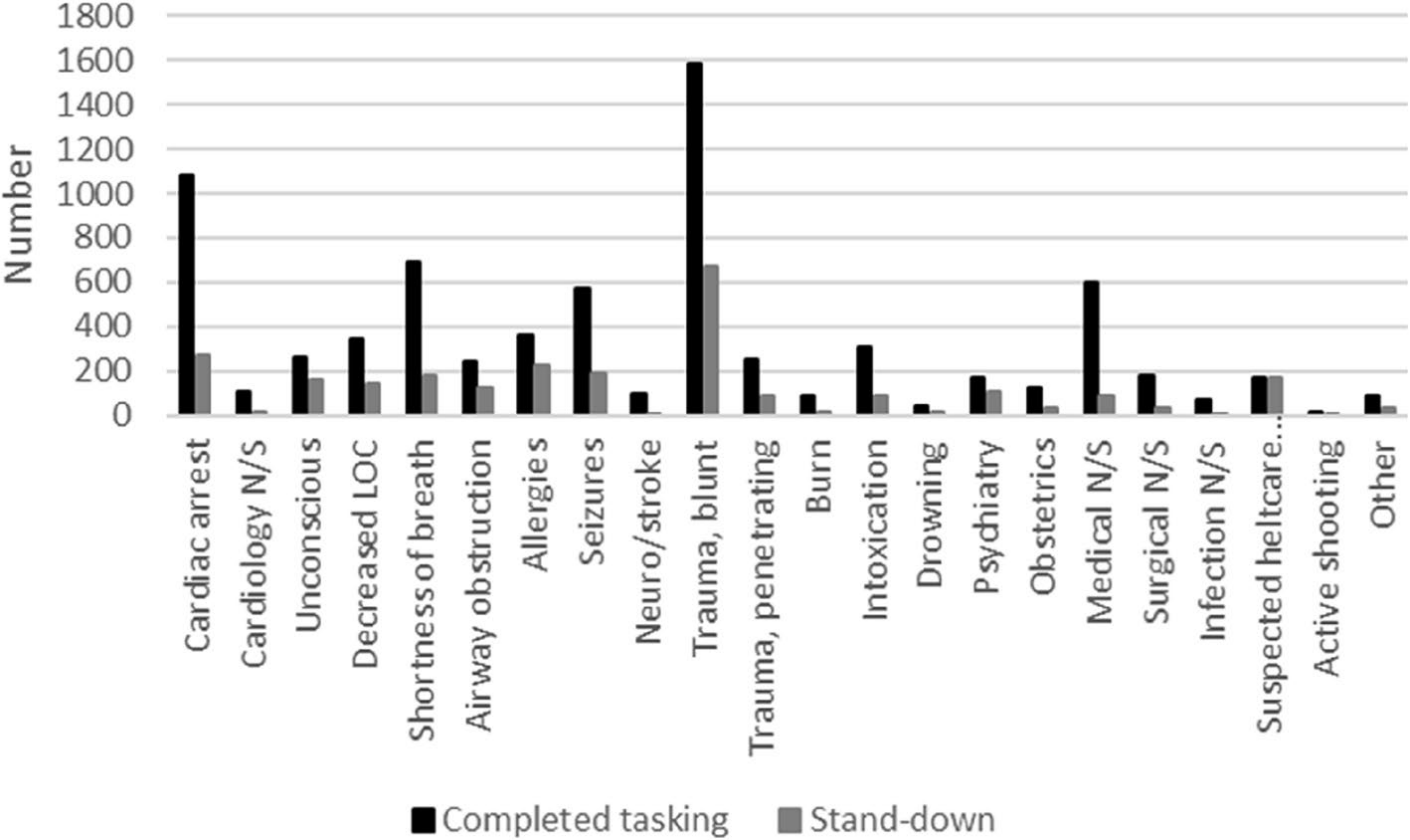
Dispatch categories: completed tasking and stand-downs (in numbers)

In 2021, RRVs in Stockholm were dispatched on 11,283 occasions, of which 3,571 (31.6%) resulted in stand-downs (Table 1). In general, stand-downs were less frequent for older patients. The most common dispatch category was blunt trauma (1,584 or 14.0%), which accounted for the highest frequency of stand-downs (676 or 6.0%). The second most common category was cardiac arrest (1,086 or 9.6%), followed by shortness of breath (691 or 6.1%), medical N/S (596 or 5.3%), and seizures (572 or 5.1%) (Fig. 1).

**Table 1:** Completed tasking and stand-downs during 2021: Rapid response cars in Stockholm

	Completed tasking	Stand-downs	*p*-value
Total number (%)	7,712 (68.4%)	3,571 (31.6%)	
Age, years (SD)	43.4 (29.3)	34.1 (29.4)	< 0.001
Priority 1	6,433 (84.4%)	3,424 (95.9%)	
Priority 2	963 (12.5%)	80 (2.2%)	
Priority 3	189 (2.5%)	17 (0.5%)	
Priority 4	1 (0%)	1 (0%)	
Priority 9	47 (0.6%)	1 (0%)	
NACA 0	157 (2.0%)	51 (1.4%)	
NACA I	423 (5.5%)	48 (1.3%)	
NACA II	636 (8.2%)	82 (2.3%)	
NACA III	1,935 (25.1%)	222 (6.2%)	
NACA IV	1,004 (13.0%)	61 (1.7%)	
NACA V	618 (8.0%)	20 (0.8%)	
NACA VI	628 (8.1%)	20 (0.8%)	
NACA VII	327 (4.2%)	115 (3.2%)	
Unknown NACA	1,984 (25.7%)	2,952 (82.7%)

**Fig. 2:**
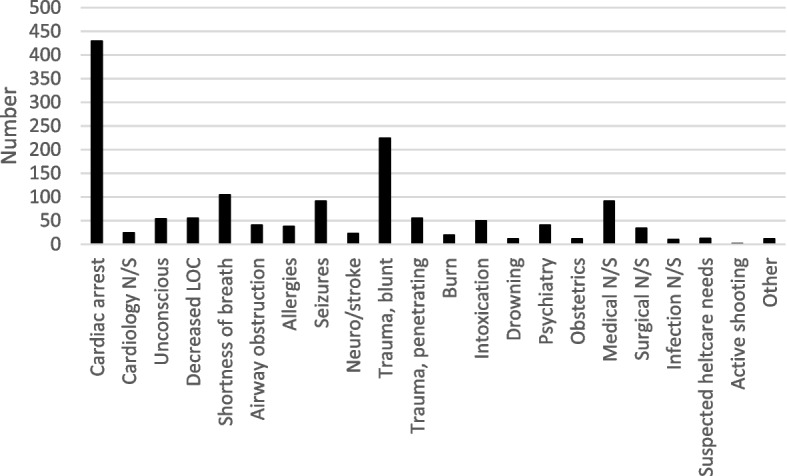
Dispatch categories, self-reported significant impact, or deviation from guidelines. Dispatch categories in which the RRV team reported that the team had a significant impact on healthcare provided to the patient or deviated from current guidelines or standard operating procedures (in numbers)

**Fig. 3:**
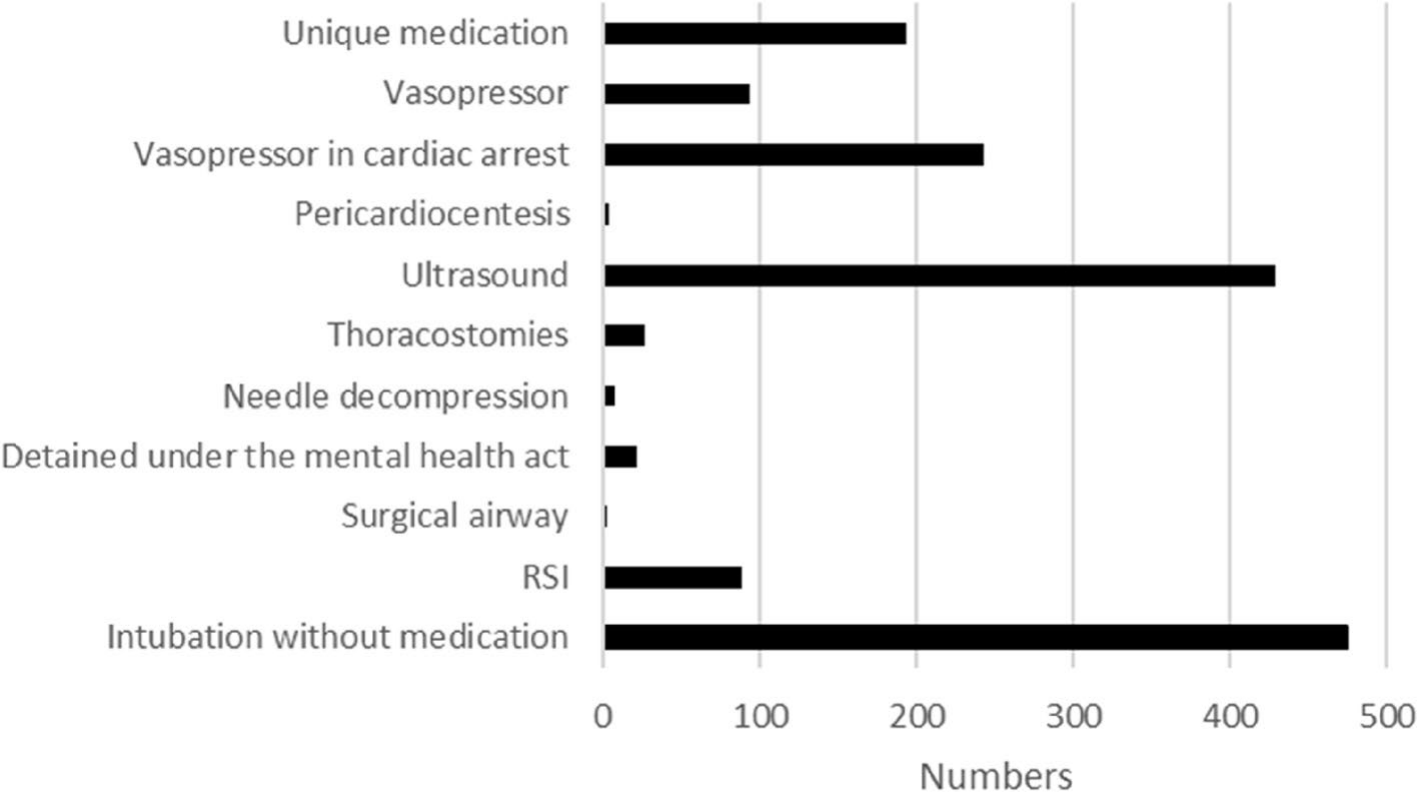
Procedures performed and administration of medications unique to the rapid response car (in numbers)

In 1,453 cases (12.9%), the RRV team reported that the team had a significant impact on healthcare provided to the patient or that they deviated from current guidelines or standard operating procedures for the patient in question (Fig. 2). Of these, the most common dispatch priority was 1 (1,247 or 85.8%), followed by priority 2 (168 or 11.6%), priority 3 (24 or 1.7%), priority 9 (8 or 0.5%), and priority 4 (1 or 0.1%). The most common NACA score was VI (283 or 19.5%), followed by NACA V (221 or 15.2%), NACA III (146 or 10.0%), NACA IV (131 or 9.0%), NACA VII (77 or 5.3%), NACA I (58 or 3.6%), and NACA II (53 or 3.6%). The ambulance on scene requested RRV support in 517/11,238 dispatches (4.6%). In 128/1,453 dispatches (8.8%), the RRV assisted with advanced pain management exceeding the ambulance guidelines. In 1,277/11,238 dispatches (11.4%), the RRV assisted with unique medication and procedures that could only be provided by RRVs (Fig. 3).

The physician or nurse in the RRV assisted with conveyance to hospital in 1,512 cases: 1,401 (92.7%) in ground ambulances and 111 (7.3%) in helicopter ambulances. Conveyance to hospital by ground ambulance was most commonly used in cases of cardiac arrest, and conveyance to hospital by helicopter ambulance was most commonly used in cases of blunt trauma. The mean NACA score was 4.7, and 14.0% of patients had no registered NACA score.

**Fig. 4:**
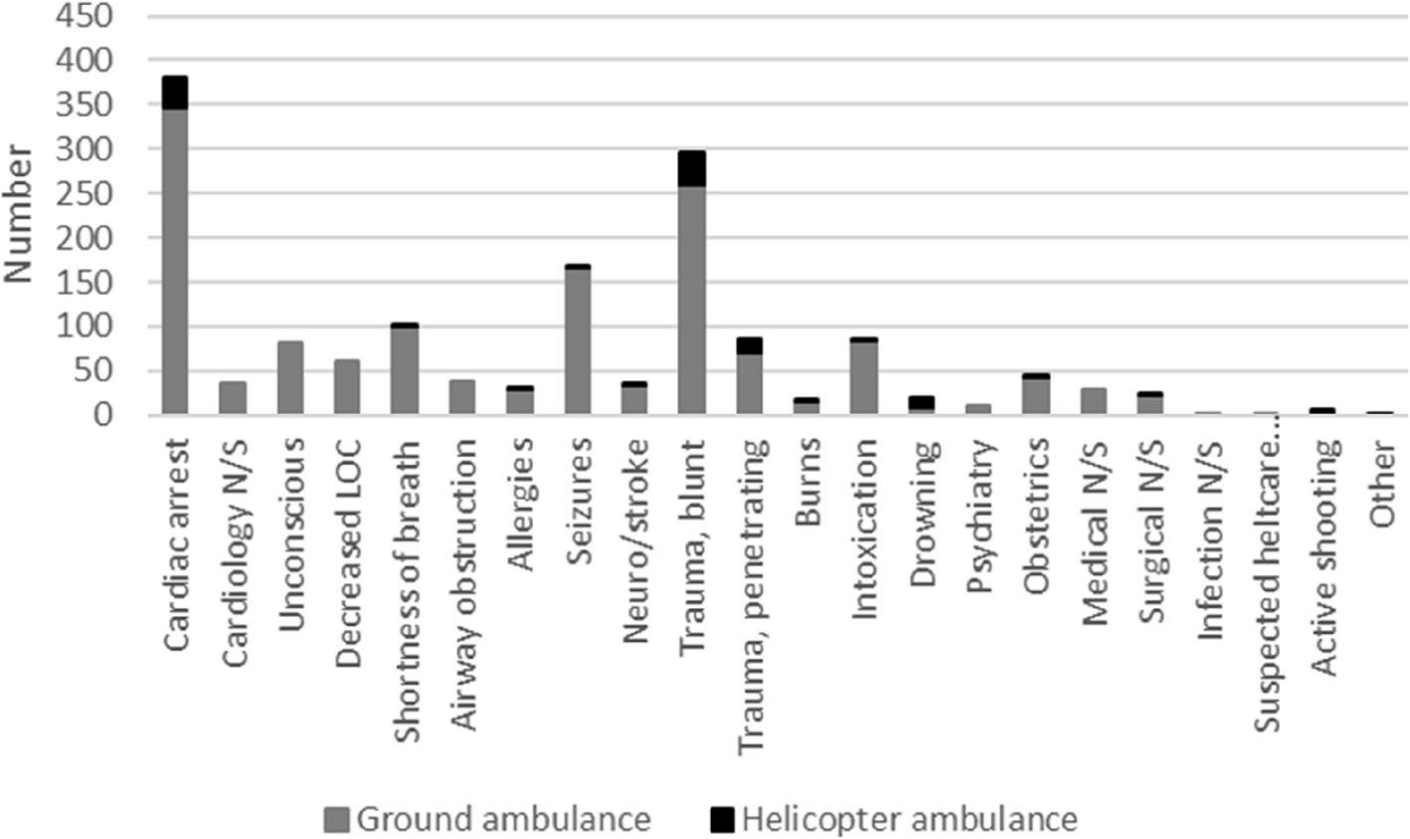
Categories of patients conveyed to hospital by ground ambulance and helicopter ambulance (in numbers)

Of the 2,601 dispatches related to trauma, 1,787 (68.7%) were registered as completed tasks, and 341 (13.1%) were registered as penetrating trauma. The most common dispatch priority was 1, accounting for 2,539/2,601 cases (97.6%). The most common NACA scores were NACA III (843/2,601 or 32.4%), followed by NACA IV (291 or 11.2%), NACA II (195 or 7.5%), NACA I (125 or 4.8%), NACA V (100 or 3.4%), NACA VI (42 or 1.6%), NACA VII (38 or 1.5%), and NACA 0 (36 or 1.4%). Of the 1,787 completed tasks, falls were the most common mechanism of trauma (666/1,787 or 37.3%), followed by traffic (579 or 32.4%) and assault (139 or 7.8%) (Fig. 4). The most common penetrating trauma mechanism was knife (144/1,787 or 8.1%), followed by gunshot (42 or 2.4%). The most frequently injured body region was the head (748/1,787 or 41.9%), followed by extremities (457 or 25.6%) and torso (434 or 24.3%).

In total, 1,357/11,238 (12.1%) dispatches related to cardiac arrest. Intubation without medication was performed in 402 (29.6%) of these cases, and RSI was performed in 12 cases (0.9%). Vasopressor medication was used in 221 cases (16.3%), and diagnostic ultrasound was used in 341 cases (25.1%).

## Discussion

The present findings confirm that RRVs in Stockholm provide valuable assistance to the ambulance service, especially in cases of cardiac arrest and trauma. RRV personnel offer the benefits of more advanced medical knowledge and can administer medications and perform interventions that the regular ambulance service cannot provide. This helps to ensure that patients receive the right care at the right time, with potentially improved outcomes.

The analysis shows that RRVs were most often dispatched to blunt trauma incidents (14%), which include fall, hanging, motor vehicle accident, and assault. The second most common category was cardiac arrest (9.8%), followed by shortness of breath (6.9%) and unclear medical problems (5.8%). While a higher number of RRV dispatches related to blunt trauma, this pattern is comparable to calls for emergency help [17–20]. The high incidence of blunt trauma was expected, as this pattern is seen across Scandinavia [21–23]. Blunt trauma cases also accounted for the greatest number of stand-downs (6%), indicating that it may be more difficult for the dispatch centre to assess whether patients in this category are severely injured.

RRV personnel most frequently reported making a significant impact in cases of cardiac arrest. In such cases, RRV personnel have specialized equipment and knowledge beyond that available in an ambulance, including a mechanical compression device, ultrasound, intubation skills, and associated drugs [7–9]. Previous studies refer to the care bundle needed to increase short- and long-term survival through return of spontaneous circulation (ROSC) [24], which may include adrenaline, oxygen, and actions for cerebral protection [25, 26]. When provided initially or within six minutes of Basic Life Support (BLS) on arrival, Advanced Life Support (ALS) is associated with increased return of spontaneous circulation (ROSC) and survival to hospital discharge [27]. We also found that a member of the RRV team most commonly accompanied ambulance patients to hospital in cases of cardiac arrest.

This further enhances patient care and treatment because RRV personnel have the ability to commence intensive care. The presence of prehospital physicians also facilitates the decision to avoid or cease cardiopulmonary resuscitation (CPR). This can prove difficult for regular ambulance personnel, as the patient’s previous medical history is available only to RRVs [4].

During the study period, the standard operating procedures (SOPs) of regular ambulances in the Stockholm region did not include tranexamic acid (TXA). Following RRV assistance, 104 patients received TXA during prehospital care. Early administration of TXA (≤ 1 h after injury) to patients in prehospital settings can increase the likelihood of survival [28]. Other RRV-specific drugs that can impact patient outcomes include magnesium sulphate for Torsades de Pointes [29, 30] or for preeclampsia and eclampsia [31]. Eclampsia is a medical emergency that requires immediate treatment to prevent both maternal and foetal mortality [32, 33], and magnesium sulphate is the first-line treatment [32, 34]. At present, regular ambulances in Stockholm do not carry magnesium sulphate; only RRVs can provide this medication and the treatments that patients with severe eclampsia or preeclampsia need. Similarly, for patients with adrenal insufficiency, a crisis can be life-threatening [35] and requires prompt treatment with hydrocortisone [36]. Again, only RRVs currently provide this medication and treatment.

Intubation without drug administration was the single most common procedure performed by RRV personnel, and this can be related to the number of cardiac arrests. As previously described, RRVs in Stockholm are staffed by at least one individual with specialist training in anaesthesiology – a nurse or physician anaesthetist. There is some evidence that on-scene time is shortened and success rate increases when prehospital tracheal intubation is performed by an experienced anaesthetist [37, 38].

Effective and timely airway management is a priority for sick and injured patients. Although the practice and benefits of prehospital emergency anaesthesia, RSI, and advanced airway management remain controversial, it is undeniable that a proportion of critically ill and injured patients require urgent advanced airway management prior to hospital arrival [39]. The European HEMS and Air Ambulance Medical Working Group (EHAC MWG) suggests that the requirement for and provision of RSI should be assessed on a case-by-case basis. Where RSI is indicated, it should be performed in a timely fashion and should not significantly delay patient transfer to hospital [39, 40].

While intubation was the most common Stockholm RRV procedure, RSI was a much rarer event. Intubation without medication occurred more than nine times each week, but RSI was performed only once or twice a week. When performing endotracheal intubation, RRV personnel typically use a video laryngoscope as a first-hand choice. This method increases first-attempt success and enhances overall intubation performance while minimizing interruption of chest compressions during CPR [41–43]. It is important to aim for a high rate of first-attempt success, as repeated attempts can result in severe complications for the patient [44, 45]. In difficult airway cases, RRVs may work together to benefit from the combined knowledge of the ambulance nurse, emergency physician, anaesthesia nurse, and anaesthesia/intensive care physician.

Ultrasound was the second most frequently used item of RRV-specific equipment. In prehospital settings, using ultrasound can change the approach to patient management, as for example in cases of cardiac arrest and termination of CPR [46–50]. Ultrasound can also predict the need for intervention in cases of trauma or breathing difficulties, but it is unclear whether this improves patient outcomes [50, 51]. During patient transport, ultrasound can be helpful when the environment makes it impossible to use a stethoscope.

As in the rest of the world, trauma is one of the leading causes of death in Sweden [52]. Trauma deaths can occur early in prehospital settings or at the hospital and are mainly a result of exsanguination or cerebral injuries [21, 22, 53]. Our analysis indicated that RRV assisted the ambulance service in caring for trauma patients almost five times each day. Most of these patients had NACA scores of III or IV, indicating severe injuries and possible deterioration of vital signs.

Haemodynamically unstable and head-injured patients benefit from rapid transport to hospital [54, 55]. Deciding what to do at the scene or en route and what to hand over to the hospital are among the most complicated decisions for everyone working in prehospital care. We believe that RRVs staffed by experienced prehospital nurses and senior consultants contribute significantly to the decision-making process.

Blunt trauma is the predominant trauma mechanism in Scandinavia, and our analysis identified this as the most common trauma mechanism in cases involving RRVs. In our study, these cases most often involved falls and traffic injuries; this was expected, as it is the typical pattern of trauma in Scandinavia [21–23]. In Sweden, there are more cases of penetrating trauma injury [56], and RRVs attend such cases about three or four times each week. As Stockholm’s ambulance service has not adopted the guidelines for traumatic cardiac arrest, such cases are treated as medical cardiac arrest unless an RRV attends the scene. We found that RRVs attended one or two traumatic cardiac arrests every week. Although survival rates are low [57, 58], traumatic cardiac arrest patients benefit from prompt and specific treatment such as thoracostomy, needle pericardiocentesis, and definitive airway management [59].

One Danish study concluded that patients benefited from more advanced prehospital intervention following the introduction of a physician-staffed helicopter; the number of patients receiving opioids also increased [60]. At present, regular Stockholm ambulances do not provide stronger opioids (such as fentanyl intravenous) and can only administer opioids in limited amounts [16]. As pain management is among the most common supports provided by the ambulance service [61], RRVs help to ensure that ambulance personnel are not limited in this regard.

Scandinavia’s various prehospital systems offer differing advantages. Although the Swedish system is based on prehospital care providers with three to five years of education, the incorporation of RRVs has additional everyday benefits for the citizens of Stockholm. For example, as compared to helicopter ambulance services in rural areas, RRVs in the more densely populated Stockholm region respond to a much higher number of dispatches, with no secondary missions [62].

The retrospective design of the present study has some limitations in terms of data quality. In particular, the information was gathered from a system with no mandatory fields for assignment registration, and as there is no provision for alerts when dispatches are not registered, assignments may be missed or inaccurately recorded. As both physicians and nurses can see the information registered, at least two people are aware of the input. However, the system does not register certain items, such as information about gender, which would be of interest for analysing gender differences in cases of trauma. Although this study refers to a limited area in Scandinavia, we believe that the data and information provided here may be of value to anyone setting up physician-manned prehospital units elsewhere. Although it is difficult to specify the difference that experience and medical training can make in critical care settings, we believe the present findings highlight some of the benefits of physician-staffed prehospital units.

## Conclusion

The present findings confirm that RRVs provide valuable assistance to ambulance services in Stockholm, especially for cardiac-arrest and trauma patients. RRVs offer the advantages of more advanced medical knowledge and can administer medication and perform interventions that the regular ambulance service cannot provide. The information provided here is likely to be of value in setting up physician-staffed prehospital units.

## Supplementary Information

**Additional file 1: Appendix 1.** The quality data collection system captures the following parameters.

## Data Availability

The ethical approval stipulates that data generated and/or analysed during this study are not publicly available and can only be distributed to the research group. However, reasonable requests for further information can be addressed to the corresponding author.

## Abbreviations

ALS: Advanced life support
BLS: Basic life support
CPR: Cardiopulmonary resuscitation
EHAC MWG: European HEMS and Air Ambulance Committee Medical Working Group
EMT: Emergency Medical Technician
NACA: National Advisory Committee for Aeronautics
N/S: Not specified
PHTLS: Prehospital trauma life support
ROSC: Return of spontaneous circulation
RRV: Rapid response vehicle
RSI: Rapid sequence induction
SBAR: Situation, Background, Assessment, Recommendation
SD: Standard deviation
SOP: Standard operating procedures
TXA: Tranexamic acid
