# Supplementary material for: Physician-staffed prehospital units: a retrospective follow-up from an urban area in Scandinavia
Source: Int J Emerg Med. 2023 Jul 14;16:43. doi: 10.1186/s12245-023-00519-8 (PMC10349430; doi:10.1186/s12245-023-00519-8)
Supplement: Supplementary file 1 — Additional file 1: Appendix 1. The quality data collection system captures the following parameters. [file 12245_2023_519_MOESM1_ESM.docx]

**Appendix 1**

The quality data collection system captures the following parameters:

Age (in years);

Priority (1, 2, 3, 4, 5, or 9).

Priority 1 means *very urgent assignment, life-threatening condition*. Priority 2 means *urgent assignment, acute condition, non-life-threatening*. Priority 3 means *non-urgent assignment, non-acute condition, no impact on patient for waiting*. Priorities 4–9 indicate that the patient needs assessment for a non-urgent or non-life-threatening condition. Priorities 5 and 9 typically include a visit from a nurse or family doctor.

Each patient is assigned a National Advisory Committee for Aeronautics (NACA) rating as follows; NACA 0: no injury/disease; NACA I: minor injury/disease – no treatment needed; NACA II: injury/disease requires examination but not hospital admission; NACA III: injury/disease without acute threat to life but requiring hospital admission; NACA IV: injuries/diseases possibly leading to deterioration of vital signs; NACA V: injuries/diseases with acute threat to life; NACA VI: CPR; NAVA VII: patient deceased.

Registration includes operational data regarding the following variables: Cardiac arrest, Cardiology N/S, Unconscious, Decreased LOC, Shortness of breath, Airway obstruction, Allergies, Seizures, Neuro/stroke, Trauma blunt, Trauma penetrating, Burn, Intoxication, Drowning, Psychiatry, Obstetrics, Medical N/S, Surgical N/S, Infection N/S, Suspected healthcare needs, Active shooting, Other.

Injured body region: head, torso, extremity.

Type of trauma: traffic injuries, fall, assault, hanging, drowning, smoke inhalation, hypothermia, other.

Weapon used: knife, gun, other.

Interventions performed by RRV include the following: rapid sequence induction (RSI), intubation without any sedative medication, vasopressor during cardiac arrest, vasopressor other, advanced pain relief (dosage other than ambulance guidelines). Intubation without medication can be performed by some prehospital nurses (i.e. trained anaesthesia nurses). RSI can only be performed by RRVs and ambulance helicopters in Stockholm.

The RRV team may also report that the team had a significant impact on healthcare provided or that it deviated from current guidelines or standard operating procedures for the patient in question.

RRVs carry specilized equipment not found in regular ambulances, including a videolaryngoscope (MacGrath), ultrasound (Butterfly), and a mechanical chest compression device (LUCAS). RRVs also carry medications not found in regular ambulances, including magnesium, oxytocin, hydrocortisone, flumazenil, tranexamic acid, sodium chloride, calcium gluconate, and hydroxocobalamin.

*Stand-downs*

If ambulance personnel decide they do not need assistance from an RRV, they make radio contact to report the patient’s SBAR status (Situation, Background, Assessment, Recommendation). Following an ambulance report that further assistance is not needed, the RRV physician makes a decision to abort the assignment or proceed
